# Integrated Plasma and Tissue Lipid Profiling Demonstrates a Distinctive Metabolic Profile in MAFLD-Associated Non-Cirrhotic Hepatocellular Carcinoma

**DOI:** 10.3390/ijms27136060

**Published:** 2026-07-06

**Authors:** Fatema Safri, Russell Pickford, Yikun Xu, William Yang, Romario Nguyen, Lawrence Yuen, Vincent Lam, Christopher Nahm, Tony Pang, Jacob George, Liang Qiao

**Affiliations:** 1 Storr Liver Centre, The Westmead Institute for Medical Research, Westmead, Sydney, NSW 2145, Australia; fsaf0214@uni.sydney.edu.au (F.S.);; 2 Faculty of Medicine and Health Sciences, The University of Sydney, Sydney, NSW 2050, Australia; 3 Bioanalytical Mass Spectrometry Facility, The University of New South Wales, Sydney, NSW 2052, Australia; 4 Department of Upper GI and HPB Surgery, Westmead Hospital, Westmead, NSW 2145, Australia

**Keywords:** hepatocellular carcinoma, fatty liver, MAFLD, non-cirrhotic MAFLD, cirrhosis

## Abstract

Metabolic dysfunction-associated fatty liver disease (MAFLD) is now the leading cause of hepatocellular carcinoma (HCC) globally. HCC surveillance is currently restricted to patients with cirrhosis, leaving those without cirrhosis, who present with more advanced disease and poorer outcomes without adequate risk stratification tools. This study compared lipid profiles across MAFLD and MAFLD-related HCC (MAFLD-HCC) patients, with and without cirrhosis, to characterise metabolic dysregulation underlying non-cirrhotic MAFLD-HCC (*nc*MAFLD-HCC). Plasma and liver lipidomic profiles were obtained from 221 patients (140 MAFLD, 66 cirrhotic MAFLD-HCC (*c*MAFLD-HCC), and 15 *nc*MAFLD-HCC) using untargeted liquid chromatography mass spectrometry. Univariate, multivariable and enrichment analyses were performed for statistically determining the lipid profile difference between the groups. Seventy percent of lipid classes were more abundant in MAFLD than in ncMAFLD-HCC and cMAFLD-HCC. Multivariate analysis revealed distinct lipid profiles across the three groups in both plasma and liver. Over 100 lipid species including diglyceride (DAG), sphingomyelin (SM), triglyceride (TG), dihydroceramide (DHCer), and linoleic acid derivatives were differentially expressed in ncMAFLD-HCC versus MAFLD, with enrichment in pathways such as glycerolipid metabolism, G-protein signalling, MAPK signalling, EGFR-TKI resistance pathway, implicated in HCC development. *nc*MAFLD-HCC exhibits a distinct lipid signature, offering preliminary mechanistic insight and a foundation for non-invasive biomarker development.

## 1. Introduction

Hepatocellular carcinoma (HCC) is a major contributor to cancer incidence globally and the third leading cause of cancer-related mortality [1]. The risk factors for HCC include viral aetiologies such as hepatitis B, hepatitis C, and hepatitis D superinfections, and alcohol-related liver disease. However, a significant epidemiological shift has occurred in recent years with non-viral aetiologies like fatty liver disease emerging as a dominant driver [2,3]. The nomenclature for fatty liver disease has undergone sequential revision, from non-alcoholic fatty liver disease (NAFLD) to metabolic dysfunction-associated fatty liver disease (MAFLD) [4], and most recently to metabolic dysfunction-associated steatotic liver disease (MASLD) [5], following a multi-society Delphi consensus. Population-level studies report substantial concordance of >90% between NAFLD/MAFLD and MASLD cohorts, indicating that findings from MAFLD-based studies remain largely applicable under the revised framework [6,7,8]. We retain MAFLD terminology throughout for consistency with the original cohort classification at the time of recruitment.

MAFLD prevalence is rising due to the global rise in metabolic disorders such as obesity and type 2 diabetes [9,10,11]. Of particular concern, fatty liver disease is implicated not only as a primary aetiology for HCC but also as a comorbid factor in nearly half of all HCC cases [12]. MAFLD-related HCC (MAFLD-HCC) demonstrates unique clinical characteristics, with ~40% of the cases developing in the absence of cirrhosis [13,14]. Published studies [15,16,17] including our own [18] have suggested that the non-cirrhotic subset is often diagnosed incidentally, and patients frequently present at advanced tumour stages with greater chances of recurrence, contributing to poorer clinical outcomes and reduced overall survival.

In metabolic liver diseases, disruption of lipid metabolism is often associated with disease stage. Lipid reprogramming, which alters lipid uptake, storage, and metabolism in the liver, is involved in the progression of MAFLD to HCC [19,20], with increased levels of triglyceride and free fatty acids in liver also linked to HCC [21,22]. However, how the lipid-rich environment in MAFLD aids the development of HCC, especially in the populations without cirrhosis, remains unexplored. There is thus a need to understand the development of HCC in this population and to develop biomarkers of HCC risk that can inform cost-effective surveillance strategies. High-throughput approaches such as metabolomics and lipidomics, in combination with transcriptomics and proteomics, can aid in this process [23]. Research develops biomarkers for various cancers, including liver cancer, has leveraged these untargeted techniques [24,25,26].

In this study we utilised blood and tissue samples from well-characterised MAFLD patients with and without HCC to identify the key signalling pathways influencing the progression to HCC. Using untargeted lipid profiling in plasma and liver, we were able to identify altered lipid classes in non-cirrhotic MAFLD-related HCC (*nc*MAFLD-HCC) and cirrhotic MAFLD-related HCC (*c*MAFLD-HCC) and their association with metabolic pathways involved in cancer development. These findings provide insights into lipid-mediated metabolic dysregulation and their potential for risk stratification, diagnosis and treatment.

## 2. Results

### 2.1. Study Workflow and Clinical Characteristics of the Study Population

**Figure 1:**
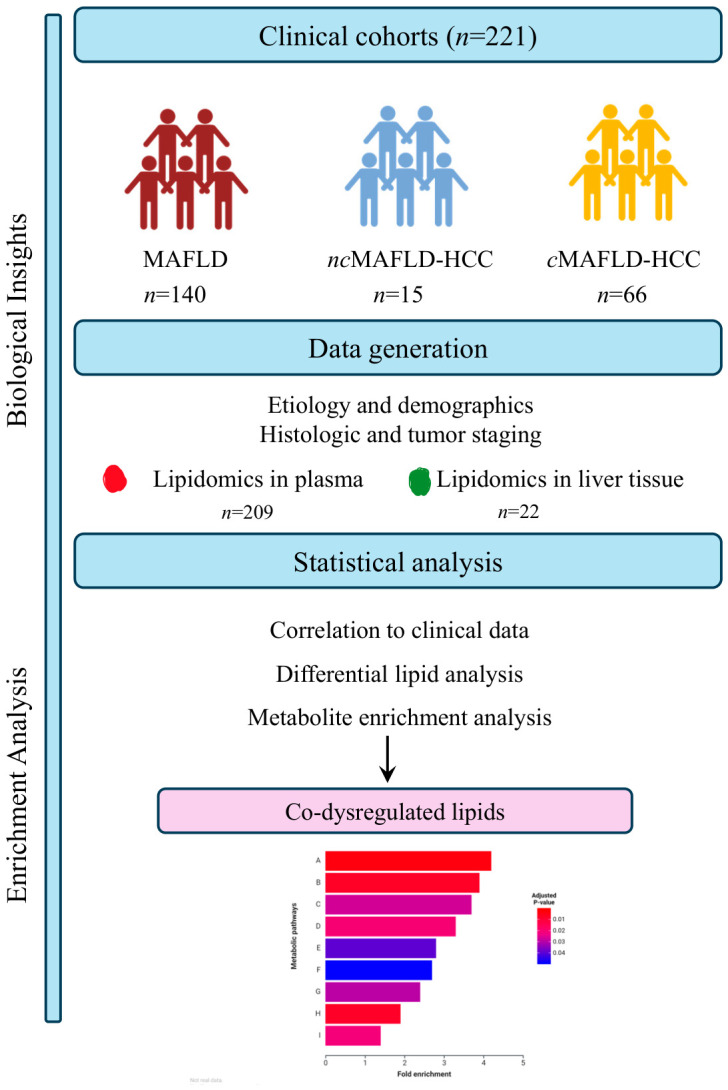
Study design and workflow.

The study population consisted of the following three cohorts: (i) MAFLD (*n* = 140); (ii) *c*MAFLD-HCC (*n* = 66); and (iii) n*c*MAFLD-HCC (*n* = 15). A total of 209 plasma samples and 22 liver tissue samples were used for lipid profiling. The relative abundance of lipids in plasma and liver tissue was assessed. Combined analysis on the plasma and liver lipids was also performed to understand the profiles within the liver and blood. Using the differential lipid species, enrichment analyses on the lipid-associated metabolic pathways were performed to highlight the significant pathways associated with the development of HCC in the non-cirrhotic population. The study design and workflow of data analysis is illustrated in Figure 1.

The baseline characteristics of the patients in the three categories of patients are reported in Table 1. Both *nc*MAFLD-HCC and *c*MAFLD-HCC patients were older compared to the MAFLD only population. Notably, over 35% of individuals with MAFLD-HCC had hypertension, and the prevalence of diabetes was twice as high in this group relative to patients without HCC, likely an age effect. Among the three groups, serum levels of triglycerides (TGs), total cholesterol, low-density lipoprotein (LDL), and high-density lipoprotein (HDL) were highest in *nc*MAFLD-HCC group, followed by MAFLD and then *c*MAFLD-HCC. As expected, MAFLD patients exhibited higher mean platelet counts, serum albumin levels, and lower FIB-4 scores compared to both HCC subgroups.

Clinical and tumour characteristics of *nc*MAFLD-HCC and *c*MAFLD-HCC patients are summarised in Table 2. Patients with *nc*MAFLD-HCC presented with more advanced BCLC stages compared to those with *c*MAFLD-HCC. Over 60% of *nc*MAFLD-HCC patients exhibited major branch vascular thrombi versus 15% in the *c*MAFLD-HCC group. The *nc*MAFLD-HCC cohort tended to have a greater number of lesions and larger tumour sizes, *albeit* this was not significant. Smoking prevalence and serum platelet counts were significantly higher in *nc*MAFLD-HCC patients compared to *c*MAFLD-HCC patients, findings consistent with other reports [17,27].

### 2.2. ncMAFLD-HCC Patients Show a Distinctive Lipid Profile in Plasma

A total of 1,293 lipid species spanning 30 different lipid classes were identified in the plasma from MAFLD, *nc*MAFLD-HCC, and *c*MAFLD-HCC cohorts using LC-MS/MS (Figure S2a). All lipid classes except trihexosylceramide (Hex3Cer), simple Glc series (CerG3GNac1), lysophosphatidylcholine (LPC), and sterols (ST) showed a similar trend with their relative abundance reducing from MAFLD to *nc*MAFLD-HCC to *c*MAFLD-HCC (Figure S3).

**Figure 2:**
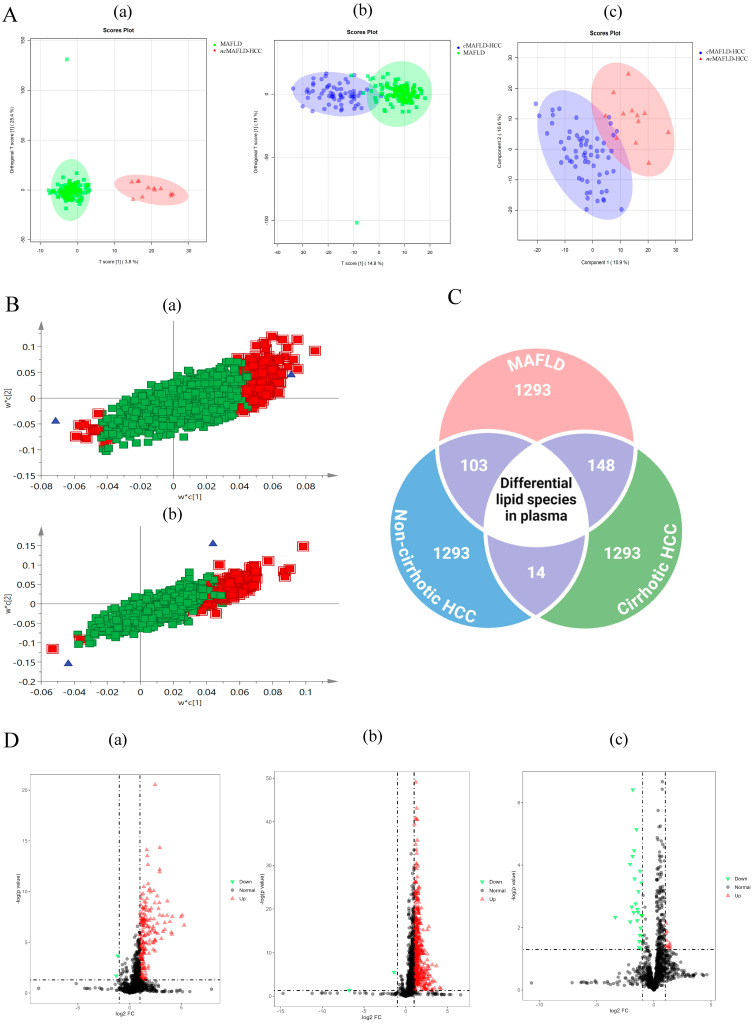
Lipid profiles in plasma samples of MAFLD and HCC cohorts. (**A**). OPLS-DA model showing the distinct separation of lipid profiles between MAFLD and *nc*MAFLD-HCC (**a**), MAFLD and *c*MAFLD-HCC (**b**), and *c*MAFLD-HCC and *nc*MAFLD-HCC (**c**); (**B**). loading plots for lipid profiles between *nc*MAFLD-HCC and MAFLD (**a**), and between *c*MAFLD-HCC and *nc*MAFLD-HCC (**b**); (**C**). Venn diagram depicting differential lipid species across each group; (**D**). volcano plots showing differential lipid species in MAFLD vs. *nc*MAFLD-HCC (**a**), MAFLD vs. *c*MAFLD-HCC (**b**) and *c*MAFLD-HCC vs. *nc*MAFLD-HCC (**c**). OPLS-DA: orthogonal partial least squares-discriminant analysis.

Unsupervised principal component analysis (PCA) (Table S2) and orthogonal partial least squares-discriminant analysis (OPLS-DA) revealed a distinct separation of the *nc*MAFLD-HCC plasma lipidome from both MAFLD and *c*MAFLD-HCC cohorts (Figure 2A, B), indicating a distinct lipidomic signature. Differential analysis of *nc*MAFLD-HCC and *c*MAFLD-HCC (Figure 2(Da–c)) showed that 14 lipid species are distinct (*p* < 0.05) (Table S3). Among these, PC (17:0_12:0), PI (16:0_16:0), TG (16:0_11:1_14:0), and TG (16:0_14:1_16:0) were downregulated, while LPI (20:4), SM (d14:0_24:5), PE (16:0_22:6), PE (18:0_20:5), SM (d40:4), CerP (d44:8), PE (18:1_22:6), PC (39:6), PC (17:0_20:5) and TG (18:1_18:1_22:6) were upregulated in *nc*MAFLD-HCC cohort. These lipid species belong to the classes sphingomyelin (SM), phosphatidylcholine (PC), ceramide phosphate (CerP), phosphatidylethanolamine (PE), phosphatidylinositol (PI), triglyceride (TG) and lysophosphoinositol (LPI).

Further comparison of the lipid profile between MAFLD and *nc*MAFLD-HCC cohorts revealed 103 lipid species that are significantly distinct (*p* < 0.05) (Figure 2C), belonging to diacylglycerol (DG), PC, LPC, ceramide (Cer), TG, and SM classes (Table S4). The MAFLD cohort showed higher level of all lipids except for PC (26:0_10:2), compared to the *nc*MAFLD-HCC cohort.

To investigate lipid dysregulation across disease progression, lipidomic profiles of MAFLD and *c*MAFLD-HCC cohorts were compared. As anticipated, there was a marked reduction in the relative abundance of lipids in *c*MAFLD-HCC patients compared to MAFLD alone patients. A total of 148 lipid species were significantly altered between the two groups (Table S5), primarily belonging to LPC, PC, DAG, Cer, TG, cholesteryl esters (ChE), PE, and SM classes.

### 2.3. ncMAFLD-HCC Patients Showed a Distinctive Lipid Profile in Liver

**Figure 3:**
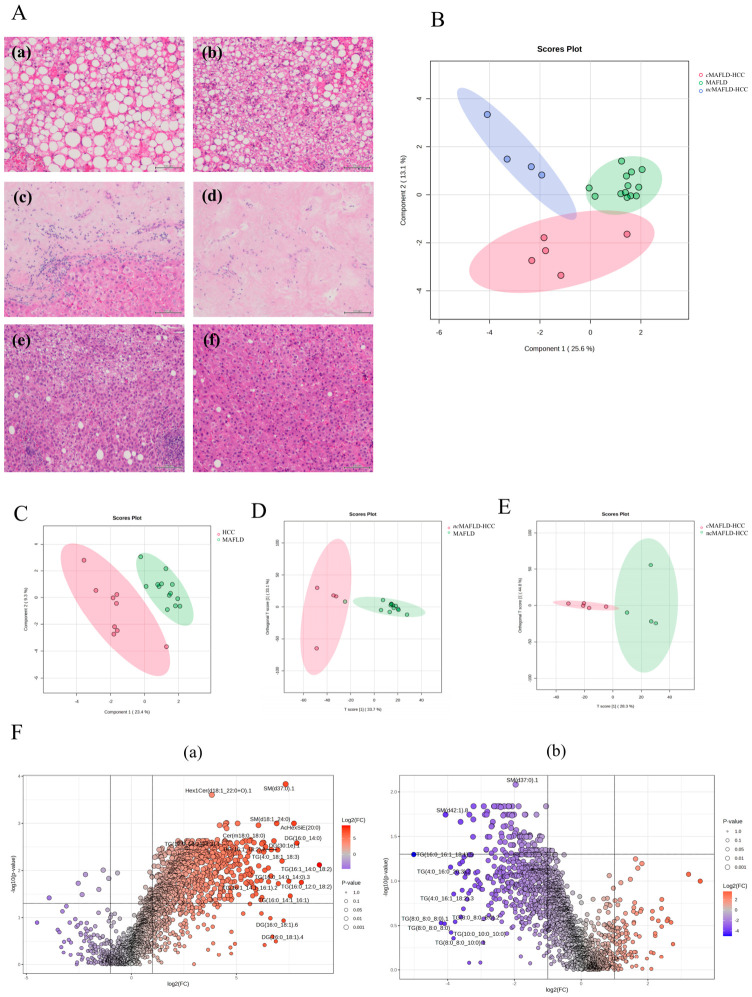
Lipid profiles in tissue samples of MAFLD and HCC cohorts. (**A**). Representative liver histology of the MAFLD (**a**,**b**), *c*MAFLD-HCC (**c**,**d**) and *nc*MAFLD-HCC (**e**,**f**) groups. H and E stain, magnification: 100×. (**B**). Distinct lipid profiles across all three groups (OPLS-DA analysis); (**C**). distinct lipid profiles between MAFLD and HCC; (**D**). distinct lipid profiles between MAFLD and *nc*MAFLD-HCC; (**E**). distinct lipid profiles between *c*MAFLD-HCC and *nc*MAFLD-HCC; (**F**). volcano plot for lipid profiles in MAFLD vs. *nc*MAFLD-HCC (**a**) and HCC vs. MAFLD (**b**). Graphs are plotted as log of the *p* value versus fold change (FC). A FC > 2 and a *p* < 0.05 is considered statistically significant. Red: up-regulation in MAFLD; blue: down-regulation in HCC. Top 20 significant lipid species are labelled in the graphs.

To investigate lipid alterations at the tissue level, liver samples from MAFLD and HCC cohorts were analysed using LC-MS/MS and histological staining. Histological assessment of liver (Figure 3A) revealed a progressive decline in lipid accumulation from MAFLD to *c*MAFLD-HCC. Multivariable OPLS-DA analysis demonstrated a clear separation in lipid profiles among the three groups (Figure 3B–E).

In total, 3458 lipid species spanning 20 lipid classes were detected across liver tissues from the MAFLD and HCC cohorts (Figure S2b). Of these, 820 lipid species were uniquely altered in the *nc*MAFLD-HCC group compared to MAFLD only patients. These lipid species predominantly included lipids from Cer, DAG, Hex1Cer, Hex2Cer, LBPA, LPC, LPG, PC, PE, PG, SM, and TG classes (Figure 3(Fa)), with additional contributions from Co, ChE, LPE, PI, PS, and SPH (Table S6). Due to the limited sample size, statistical comparison between the *nc*MAFLD-HCC and *c*MAFLD-HCC groups was not feasible; however, a noticeable trend of decreased lipid abundance in *c*MAFLD-HCC liver was observed.

Comparison between HCC (cirrhosis and non-cirrhosis) and the MAFLD group identified 142 significantly dysregulated lipid species (*p* < 0.05) in HCC (Figure 3(Fb)), predominantly belonging to SM, TG, Cer, PG, PE, and PC classes (Table S7).

### 2.4. Co-Dysregulated Lipid Profile of Plasma and Tissue in the ncMAFLD-HCC Cohort

**Figure 4:**
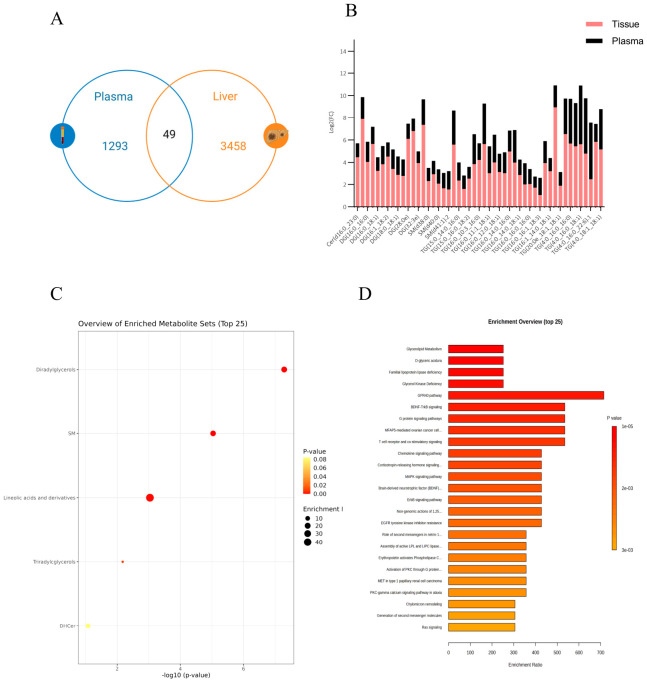
Co-dysregulated lipid species in the plasma and liver of the *nc*MAFLD-HCC cohort. (**A**). Venn diagram representing the common dysregulated lipid species in plasma and liver tissue; (**B**). bar graph representing all significant co-dysregulated lipid species in plasma and liver in MAFLD and *nc*MAFLD-HCC cohorts (FC > 2; *p*^adj^ < 0.05). Red bars: log FC in liver; black bars: log FC in plasma. (**C**). Enriched lipid classes based on the significant co-dysregulated lipid species. The size of the dots represents the enrichment ratio (based on the hits), and the colour of the dots indicates the statistical significance (based on *p* values); (**D**). pathway enrichment analysis of co-dysregulated lipid species from Relational Database of Metabolomics Pathways (RaMP-DB). The colour darkness of the bars represents the significance, based on the *p* values, and the bar length represents the enrichment ratio (hits/expected).

To gain insights into lipid dysregulation in the *nc*MAFLD-HCC cohort, a combined analysis of plasma and liver lipid profiles from the MAFLD and *nc*MAFLD-HCC groups was performed. This integrative approach identified 49 lipid species that were significantly co-dysregulated in both compartments, spanning multiple lipid classes (Figure 4A). These species were more abundant in the MAFLD cohort compared to the *nc*MAFLD-HCC, indicating reduced lipid levels in the cancerous liver (Table S8). Notably, fold change comparisons revealed greater dysregulation in liver than in plasma for the majority of the species except triglycerides such as TG (4:0_16:0_22:6) and TG (16:0_16:1_18:3) (Figure 4B).

**Figure 5:**
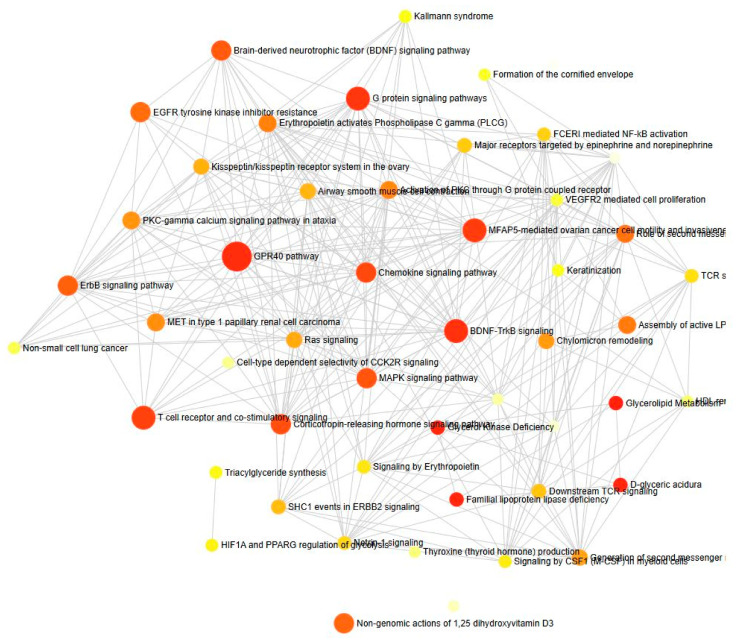
Network visualisation of the most significant metabolic pathways based on the co-dysregulated lipid species. Lipid species clustering into lipidomic pathways. Each dot represents the metabolic pathways, where the size of the circle represents the significance of that pathway based on *p* values, and the intensity of the colour of the dots represents the up or downregulation according to the fold changes.

Structural classification of the co-dysregulated lipids showed that most of them belonged to DAG, SM, linoleic acid and its derivatives, TG and dihydroceramides (DHCer) (Figure 4C). To explore the biological relevance of these alterations, metabolite set enrichment analysis (MSEA) was conducted using RaMP-DB version 2.0, which integrates data from KEGG, HMDB, Reactome, and WikiPathways. This revealed significant enrichment of metabolic pathways such as glycerolipid and sphingolipid metabolism, as well as signalling pathways involving DAG (diacylglycerols) and IP3 (inositol trisphosphate). Additionally, pathways associated with G-protein signalling, the GPR40 pathway, BDNF-TrkB signalling, ErbB signalling pathway, MAPK signalling, epidermal growth factor receptor (EGFR) tyrosine kinase inhibitor resistance, and Ras kinase were enriched in the *nc*MAFLD-HCC cohort. The top 25 enriched pathways are presented in Figure 4D, with significantly enriched pathways (*p* < 0.05) listed in Table S9. A network representation of lipid involvement across these pathways is shown in Figure 5.

## 3. Discussion

Using an integrative lipidomics approach on plasma and liver, this exploratory study profiled over 1200 lipid species in plasma and more than 3400 in liver, providing one of the first comprehensive characterisations of lipid alterations in MAFLD-associated non-cirrhotic HCC. The innovative aspect of this study lies in its specific focus on ncMAFLD-HCC, a clinically important but under-characterised subgroup, and its direct comparison with both MAFLD-only patients and cirrhotic MAFLD-HCC cases. By excluding viral- and alcohol-related liver disease, this study minimises major aetiological confounders and enables a more focused assessment of lipidomic changes associated with MAFLD-related hepatocarcinogenesis. This study identifies distinct clinical phenotypes, tumour characteristics, and lipidomic signatures in *nc*MAFLD-HCC compared to both MAFLD only (i.e., without HCC) and *c*MAFLD-HCC.

We observed a progressive reduction in overall lipid abundance from MAFLD to *nc*MAFLD-HCC to *c*MAFLD-HCC, reflecting declining lipid accumulation with advancing disease stage. A variance-based clustering approach demonstrated clear separation of lipid species between groups, underscoring the uniqueness of the lipid profile in *nc*MAFLD-HCC. In plasma, lipid species from the PC, SM, Cer, DAG, and TG classes were among the most significantly altered. In the liver, a larger number of lipid species were detected, consistent with the circulating profile. Key lipid classes SM (61.2%), DAG (12.3%), TG (24.5%), and Cer (2%), were consistently altered in both plasma and liver in *nc*MAFLD-HCC. These classes are associated with some of the key signalling pathways (MAPK, EGFR tyrosine kinase inhibitor, BDNF-TrkB, and Ras signalling, and GPR40) known to play a role in oncogenesis [28,29,30,31]. Our observations are consistent with studies employing Liquid Chromatography Quadrupole Time-of-Flight Mass Spectrometry (LC-QTOFMS) and High-Performance Liquid Chromatography (HPLC) which reported lipid alterations in the serum and liver of MAFLD and MASH (metabolic dysfunction-associated steatohepatitis) patients [32,33]. Elevation of saturated TGs and decreased LPCs and sphingolipids has also been reported during the progression from NASH to HCC. In sum, this supports the notion that distinct lipid reprogramming occurs in *nc*MAFLD-HCC and the imbalance of these lipid species might play a pathogenic role in HCC development. These findings should be interpreted as exploratory and hypothesis-generating, offering preliminary insights into MAFLD-related hepatocarcinogenesis and supporting future mechanistic and biomarker-focused studies in patients with *nc*MAFLD-HCC.

Previous studies, including our own, have consistently demonstrated that patients with *nc*MAFLD-HCC tend to present with more advanced disease stage at diagnosis [18,34]. In line with this, tumour characteristics in our cohort showed a similar pattern. This likely reflects the lack of routine surveillance in non-cirrhotic populations as per current practice guidelines, meaning that diagnosis is typically delayed. An understanding of the clinical and/or biochemical predictors of those at high risk of HCC development, such as the lipidomics differences we report in the population without cirrhosis, is thus a step forward.

The trend of reduced lipid levels, both in plasma and liver of the *c*MAFLD-HCC cohort, is likely multifactorial, including reduced or lost functional capacity to synthesise, process, and export lipids [35,36,37], malabsorption of dietary fat, as well as increased consumption of fat stores to meet energy demands or immunity [38,39,40]. We report that *nc*MAFLD-HCCs also displays significant lipidomic remodelling, despite having well-preserved liver architecture and function. This suggests that the altered lipid species may actively contribute to carcinogenesis rather than merely reflecting metabolic reprogramming. Certain lipid classes such as ceramides have been implicated in protective roles against oncogenic processes through their involvement in apoptosis, membrane integrity, signalling regulation, immune response and metabolic control [41,42,43]. In this study, there was a decrease in ceramides in *nc*MAFLD-HCC as compared to MAFLD. Aberrant lipid metabolism has also been observed in lung cancer, where single-cell RNA sequencing of early-stage tumours revealed dysregulation of glycerolipid metabolism and fatty acid biosynthesis [34]. Further, an imbalance between protective and pro-tumorigenic lipids may create a permissive microenvironment for malignant transformation and progression [44].

Accumulating evidence suggests that alterations in lipid ratios may reflect metabolic reprogramming underlying hepatocarcinogenesis. For example, ratios of Cer/SM, ChE/free cholesterol and poly-/mono-unsaturated LPC is altered in the blood of HCC patients as compared to liver cirrhosis patients and normal controls [45,46]. Moreover, tissue-based analyses have reported reduced Cer levels alongside elevated ChE and SM in HCC [47,48]. In agreement with the literature, our study shows that the Cer/SM ratio is higher in MAFLD liver compared to *nc*MAFLD-HCC and significant down-regulation of SM and Cer was identified in this group. An unanswered question, however, remains as to whether the accumulation of specific lipid species drives the progression of non-cirrhotic HCC, or whether the loss of protective lipid species is the driver. Collectively, these findings reinforce that disruption of lipid homeostasis is central to hepatocarcinogenesis in *nc*MAFLD-HCC and underscores the importance of lipid ratios as a marker of disrupted metabolic homeostasis.

We acknowledge that there are limitations to this study, including that the MAFLD cohort was younger than those with HCC. Furthermore, as *nc*MAFLD-HCC is often diagnosed at advanced disease stages, liver biopsies or resections are infrequently performed, resulting in a smaller cohort with this phenotype. The smaller cohort limits the statistical exploration of the lipidomics, and further in vitro studies can delineate the underlying mechanism. Nonetheless, the consistent trends observed in both plasma and liver analyses lends robustness to the findings and support their biological relevance.

In conclusion, this study provides novel insights into the lipid dysregulation associated with *nc*MAFLD-HCC. The distinct lipidome in this group highlights their potential role in liver cancer development, and their plausibility to serve as blood biomarkers to risk-stratify patients with MAFLD who are at risk of HCC and would therefore benefit from surveillance.

## 4. Methods

### 4.1. Participants and Clinical Data Collection

We included 221 consecutive patients from Westmead Hospital, of which 140 had MAFLD as their sole liver disease and 81 had MAFLD-associated HCC. Written informed consent to obtain clinical data and biological specimens was obtained from all patients. The study was approved by the Human Research Ethics Committee of the Sydney West Area Health Service [HREC/18/WMEAD/5 (5522)].

The diagnosis of MAFLD was based on the presence of hepatic steatosis through liver biopsy or imaging (CT, MRI, or ultrasound), along with clinical evidence of metabolic disorders according to the proposed MAFLD criteria [49]. All cases of HCC were confirmed through imaging and liver histology. Patients with other underlying causes such as viral infections (Hepatitis B, C, or D), alcohol-related liver disease, hemochromatosis, or other causes of liver disease were excluded. Patients with a history of consuming more than 2 standard alcoholic drinks per day and those with unknown cirrhosis status were excluded. MAFLD patients with a METAVIR fibrosis stage of greater than 2 were also excluded. The process of patient selection using the inclusion and exclusion criteria is shown in Figure S1.

Clinical variables including patient demographics, history of metabolic disorders (such as hypertension, diabetes, and obesity), body mass index (BMI), serum triglyceride, serum cholesterol, low-density lipoproteins (LDLs), high-density lipoprotein (HDL), baseline liver function tests (LFTs), liver fibrosis score (by Fibrosis-4 Index, FIB-4) and Child–Pugh–Turcotte (CPT) scores were retrieved from the clinical records. Tumour-related information, including the number and size of tumour nodules, cirrhosis status, Barcelona Clinic Liver Cancer (BCLC) staging score, and presence of portal vein thrombosis (PVT), was collected.

### 4.2. Clinical Sample Collection and Storage

Baseline peripheral blood samples were collected from participants. Blood was drawn into EDTA tubes and processed. Samples were centrifuged at 1000× *g* for 10 min at 4 °C to isolate plasma and aliquoted into sterile cryovials and stored at −80 °C. Liver tissue was obtained intraoperatively during surgical resection. Paired samples from both the tumour and adjacent non-tumour liver were collected immediately after excision. Tissues were rinsed briefly in sterile phosphate-buffered saline to remove residual blood and stored at −80 °C until processing.

### 4.3. Histology

Paraffin-embedded liver tissue blocks were sectioned into 4 µm-thick slices and stained with Hematoxylin-eosin (H and E) to identify lipid accumulation. All slides were digitised at 100× magnification using an Olympus BX43 microscope (Tokyo, Japan).

### 4.4. Sample Preparation for Lipid Extraction

Stored plasma samples were thawed on ice. Approximately 50 mg of liver was mixed with ice-cold 1-butanol/methanol (1:1 *v*/*v*). An appropriate volume of 1-butanol/methanol (1:1 *v*/*v*) was added to normalise the tissue weight to 100 mg/mL [50,51]. The samples were homogenised twice using TissueLyser (Qiagen, Hilden, Germany) at 30 Hz for 2 min. The samples were left to cool for 1 min between the two homogenization cycles.

### 4.5. Lipid Extraction

Lipids from plasma and liver were extracted using the single-phase 1-Butanol-Methanol (BUME) method [52]. Briefly, 10 μL of plasma and 100 μL of tissue-weight normalised homogenate were aliquoted into microcentrifuge tubes. Ten μL of internal standards mixture (Table S1) and 100 μL of 1-butanol/methanol (1:1 *v*/*v*) containing 5 mM ammonium formate was added. The mixture was vortexed for 10 s, incubated for 60 min in a sonicator bath, and then centrifuged at 16,000× *g* for 10 min. The upper phase was transferred to a fresh microcentrifuge tube. The pellet was washed with 100 μL of 1-butanol/methanol (1:1 *v*/*v*) and combined with the previous extract. The samples were vacuum centrifuged until dry, resuspended in 100 μL of Isopropanol: Acetonitrile: distilled water (65:30:5 *v*/*v*) and transferred to 0.2 mL glass insert vials for analysis by Liquid Chromatography-Electrospray Ionisation-Tandem Mass Spectrometry (LC-ESI-MS/MS).

### 4.6. Liquid Chromatography with Tandem Mass Spectrometry (LC-MS/MS)

Lipid extracts (10 μL injection) were analysed using a Q-Exactive HF Mass Spectrometer (ThermoFisher Scientific, Waltham, MA, USA) coupled to a U3000 UPLC system (ThermoFisher Scientific, Waltham, MA, USA). Chromatography was performed at 60 °C on a Waters CSH C18 UHPLC column (2.1 × 100 mm), 1.8 μM with VanGuard guard column (Waters Corporation, Milford, MA, USA). Solvent A was acetonitrile: water (6:4), and Solvent B was acetonitrile: isopropanol (1:9), both containing 10 mM ammonium formate and 0.1% formic acid. Lipids were chromatographed according to the method of Castro-Perez et al. [53]. Briefly, a 30-min gradient running from 30 to 100% of solvent B was performed, eluting lipids in order of hydrophobicity. The column eluate was directed into the electrospray ionisation source of the mass spectrometer where a heated electrospray ionisation (HESI) source probe was employed. Source parameters were optimised on a range of lipid standards prior to the analysis. The mass spectrometer was run in “data-dependent acquisition” mode. A survey scan over the mass range 200–1200 at a resolution of 70 K was followed by 10 data-dependent MS/MS scans on the most intense ions in the survey at 15 K. Dynamic exclusion was used to improve the number of ions targeted. Cycle time was approximately 1 s. Samples were run in both positive and negative polarities. The samples were run in a random order (generated using Microsoft Excel) to avoid batch effects and/or changing instrument performance effects. Data were analysed in LipidSearch software 4.3 (ThermoFisher Scientific, Waltham, MA, USA). Data were searched against the standard LipidSearch database with all common mammalian lipid classes included. The search results were grouped according to sample type and aligned for differential analysis. Aligned data (containing lipid identity, retention time, and peak area) were exported to Excel software (Microsoft Corporation, Redmond, WA, USA) for further analysis. Relative abundance of lipids was obtained from peak areas normalised to internal standards.

### 4.7. Lipidomics and Statistical Analysis

For verification of data processing and analysis, statistical significance, and results, data were independently processed and analysed in the online Metaboanalyst Version 6.0 software and the lipidome analysis services at Creative Proteomics, NY, USA.

Exploratory analysis among the three cohorts was conducted on each dataset independently using the chemometrics analysis in Metaboanalyst Version 6.0 [54] and Soft Independent Modelling of Class Analogy (SIMCA). Principal Component Analysis (PCA), Partial Least Squares Discriminant Analysis (PLS-DA), and Orthogonal PLS-DA (OPLS-DA) were used to determine differences between the three groups (*c*MAFLD-HCC, *nc*MAFLD-HCC, and MAFLD). Data were filtered based on the interquartile range and mean intensity value in the Metaboanalyst software. Lipid abundance in plasma and tissue was normalised to the internal standards to help correct for analytical variation. Data were transformed to log and mean-centred for data scaling. For the differences in lipid species between groups, two-sided *t*-test and one-way analysis of variance (ANOVA) were performed in Metaboanalyst Version 6. An adjusted *p*-value (*p*^adj^) of <0.05 was considered statistically significant. Furthermore, the parameter of variable influence of projection (VIP) was evaluated for each OPLS-DA model. Finally, the lipid species with a False discovery rate (FDR) corrected *p* < 0.05, fold change > 2, and VIP > 1.5 were considered statistically significant and used for enrichment and pathway analysis. To visualise statistically significant lipid species, volcano plots using log fold change and log *p* values were generated using Metaboanalyst Version 6.0.

The data report obtained from Creative Proteomics included univariable analysis to determine the differential lipid species and multivariable analysis to determine the pathways associated with the differential lipid species. Functional and enrichment analyses were performed using Metaboanalyst Version 6.0. Pathway libraries in Kyoto Encyclopaedia of Genes and Genomes (KEGG) and Human Metabolome Database (HMDB) were used to annotate statistically significant lipid species and their classes. Pathways with at least 2 lipid classes enriched (Fisher’s exact test, *p* < 0.05) were considered statistically significant.

Clinical characteristics were analysed using SPSS Version 29. Categorical data were analysed using Fisher’s exact test and are expressed as percentages. Continuous variables are analysed using the Kruskal–Wallis test and expressed as mean ± standard deviation. A *p* < 0.05 was considered statistically significant.

## Figures and Tables

**Table 1:** Clinical characteristics of MAFLD, *c*MAFLD-HCC and *nc*MAFLD-HCC cohorts.

Characteristics	MAFLD (*n* = 140)	*nc*MAFLD-HCC (*n* = 15)	*c*MAFLD-HCC (*n* = 66)	*p* Value (MAFLD vs. *nc*MAFLD-HCC)	*p* Value (MAFLD vs. *c*MAFLD-HCC)
Age (*n* = 209)	48.7 ± 12.1	69.9 ± 9.6	68.8 ± 9.1	**<0.001**	**<0.001**
Sex (male %) (*n* = 208)	56.1	75	65.6	0.229	0.36
Ethnicity (%) (*n* = 173) Caucasian Others	51 49	58.3 41.7	57.1 42.9	**0.012**	**<0.001**
BMI (kg/m^2^) (*n* = 207)	31 ± 6.3	28.8 ± 5.4	33 ± 15.4	0.386	0.373
Hypertension (mmHg) (%) (169)	33.8	40	64.7	**<0.001**	**<0.001**
Diabetes (%) (*n* = 205)	23.8	66.7	84.1	**0.002**	**<0.001**
Platelets (10 × 10^9^/L) (*n* = 206)	253.6 ± 64.9	218.8 ± 79.1	136.6 ± 68.4	0.087	**<0.001**
Albumin (g/L) (*n* = 208)	44.6 ± 3.2	37.8 ± 10.3	35.19 ± 6.5	**<0.001**	**<0.001**
LDL (mmol/L) (*n* = 142)	3.1 ± 1.1	4.9 ± 5.8	2.3 ± 0.83	0.985	0.09
HDL (mmol/L) (*n* = 145)	1.30 ± 0.4	5.1 ± 5.5	1.28 ± 0.4	0.210	0.903
ALT (U/L) (*n* = 208)	73.2 ± 44.4	46.3 ± 37.5	70.3 ± 200.1	**0.017**	**<0.001**
AST (U/L) (*n* = 208)	48.3 ± 29.0	76.6 ± 67.5	136.6 ± 592.9	0.432	**0.04**
TG (mmol/L) (*n* = 162)	2.0 ± 1.0	4.0 ± 4.9	1.2 ± 0.5	0.757	**<0.001**
Cholesterol (mmol/L) (*n* = 160)	5.3 ± 1.1	5.7 ± 2.9	4.0 ± 1.0	0.229	**<0.001**
FIB-4 (*n* = 205)	1.1 ± 0.54	6.1 ± 14.7	6.6 ± 8.1	**<0.001**	**<0.001**

BMI: Body Mass Index; LDL: Low-density lipoprotein; HDL: High-density lipoprotein; ALT: alanine transaminase; AST: Aspartate aminotransferase; TG: Triglyceride; FIB-4: Fibrosis-4 index.

**Table 2:** Clinical and tumour characteristics of *c*MAFLD-HCC and *nc*MAFLD-HCC cohorts.

Characteristics	*c*MAFLD-HCC (*n* = 66)	*nc*MAFLD-HCC (*n* = 15)	*p* Value
Age (*n* = 76)	68.8 ± 9.1	69.9 ± 9.6	0.519
Sex (male) (*n* = 76)	65.6	75	0.140
Ethnicity (*n* = 75)			
Caucasian	57.1	58.3	0.274
Others	42.9	41.7	
BMI (*n* = 76)	33 ± 15.4	28.8 ± 5.4	0.249
Smoking (%) (*n* = 74)	36.5	81.8	**<0.001**
Diabetes (%) (*n* = 75)	84.1	66.7	0.183
Hypertension (%) (*n* = 39)	64.7	40	0.331
Platelets (10 × 10^9^/L) (*n* = 77)	136.6 ± 68.4	218.8 ± 79.1	**0.002**
Albumin (g/L) (*n* = 78)	35.19 ± 6.5	37.8 ± 10.3	0.769
LDL (mmol/L) (*n* = 13)	2.3 ± 0.83	4.9 ± 5.8	1.00
HDL (mmol/L) (*n* = 13)	1.3 ± 0.4	5.1 ± 5.5	0.236
ALT (U/L) (*n* = 78)	70.3 ± 200.1	46.3 ± 37.5	0.720
AST (U/L) (*n* = 77)	136.6 ± 592.9	76.6 ± 67.5	0.900
TG (mmol/L) (*n* = 31)	1.2 ± 0.5	4.0 ± 4.9	0.345
Cholesterol (mmol/L) (*n* = 31)	4.0 ± 1.0	5.7 ± 2.9	0.953
FIB-4 (*n* = 77)	6.6 ± 8.1	6.1 ± 14.7	**0.022**
Family history of HCC (*n* = 71)	15.3	25	**0.04**
BCLC staging (*n* = 65)			
0	12.3	0	**<0.001**
A	38.6	25	
B	26.3	0	
C	22.8	50	
D	0	25	
No. of lesions (*n* = 71)			
1–3	85.0	63.7	0.229
>3	15	36.4	
Size of the largest lesion (*n* = 74)			
<2.9 cm	47.6	18.2	0.161
3–5 cm	28.6	27.3	
> 5 cm	23.8	54.5	
Portal vein thrombus (*n* = 68)			
Minor branch	5	0	**0.011**
Major branch	15	62.5	
Tumour extent (*n* = 40)			
<50% liver	83.3	75	0.375
>50% liver	16.7	25	
CTP score (*n* = 73)			
A (5–6)	66.7	70	0.650
B (7–9)	28.6	30	
C (10–15)	1.6	0	

Categorical data are represented as percent (%) and continuous data are represented as mean± SD. Kruskal–Wallis Test was used for continuous variables and Fisher Exact Test was used for categorical variables. BMI: Body Mass Index; LDL: Low-density lipoprotein; HDL: High-density lipoprotein; ALT: alanine transaminase; AST: Aspartate aminotransferase; TG: Triglyceride; FIB-4: Fibrosis-4 index; BCLC- Barcelona Clinic Liver Cancer. Child–Turcotte–Pugh (CTP) Score was calculated using the cirrhosis mortality calculator https://www.mdcalc.com/calc/340/child-pugh-score-cirrhosis-mortality (accessed on 19 January 2026).

## Data Availability

The datasets used and analysed during the current study are available from the corresponding author on reasonable request.

## Supplementary Materials

The following supporting information can be downloaded at https://www.mdpi.com/article/10.3390/ijms27136060/s1.

## Abbreviations

The following abbreviations were used in the manuscript:

MAFLD-HCC: MAFLD-associated HCC; *nc*MAFLD-HCC: non-cirrhotic MAFLD-HCC; *c*MAFLD-HCC: cirrhotic MAFLD-HCC; FIB-4: fibrosis-4 index; BCLC: Barcelona Clinic Liver Cancer; LC-MS/MS: liquid chromatography with tandem mass spectrometry; PLS-DA: Partial Least Squares Discriminant Analysis; OPLS-DA: Orthogonal PLS-DA; TG: triglycerides; SM: sphingomyelin; PC: phosphatidylcholine; CerP: ceramide phosphate; PE: phosphatidylethanolamine; PI: phosphatidylinositol (PI), PG: phosphatidylglycerol; LPI: lysophosphoinositol; LPC: lysophosphatidylcholine; LPG: lysophosphatidylglycerol; DG, DAG: diacylglycerol; Cer: ceramide; ChE: cholesteryl esters; Hex1Cer: monohexosylceramide; Hex2Cer:dihexosylceramide; Co: coenzyme; SPH: sphingosine; GPR40: G-protein coupled receptor 40; MAPK: Mitogen-Activated Protein Kinase; EGFR: epidermal growth factor receptor.
