# Supplementary material for: Integrated Plasma and Tissue Lipid Profiling Demonstrates a Distinctive Metabolic Profile in MAFLD-Associated Non-Cirrhotic Hepatocellular Carcinoma
Source: Int J Mol Sci. 2026 Jul 6;27(13):6060. doi: 10.3390/ijms27136060 (PMC13362357; doi:10.3390/ijms27136060)
Supplement: Supplementary file 1 [file ijms-27-06060-s001.zip › Supp figures.pptx]

## Slide 1
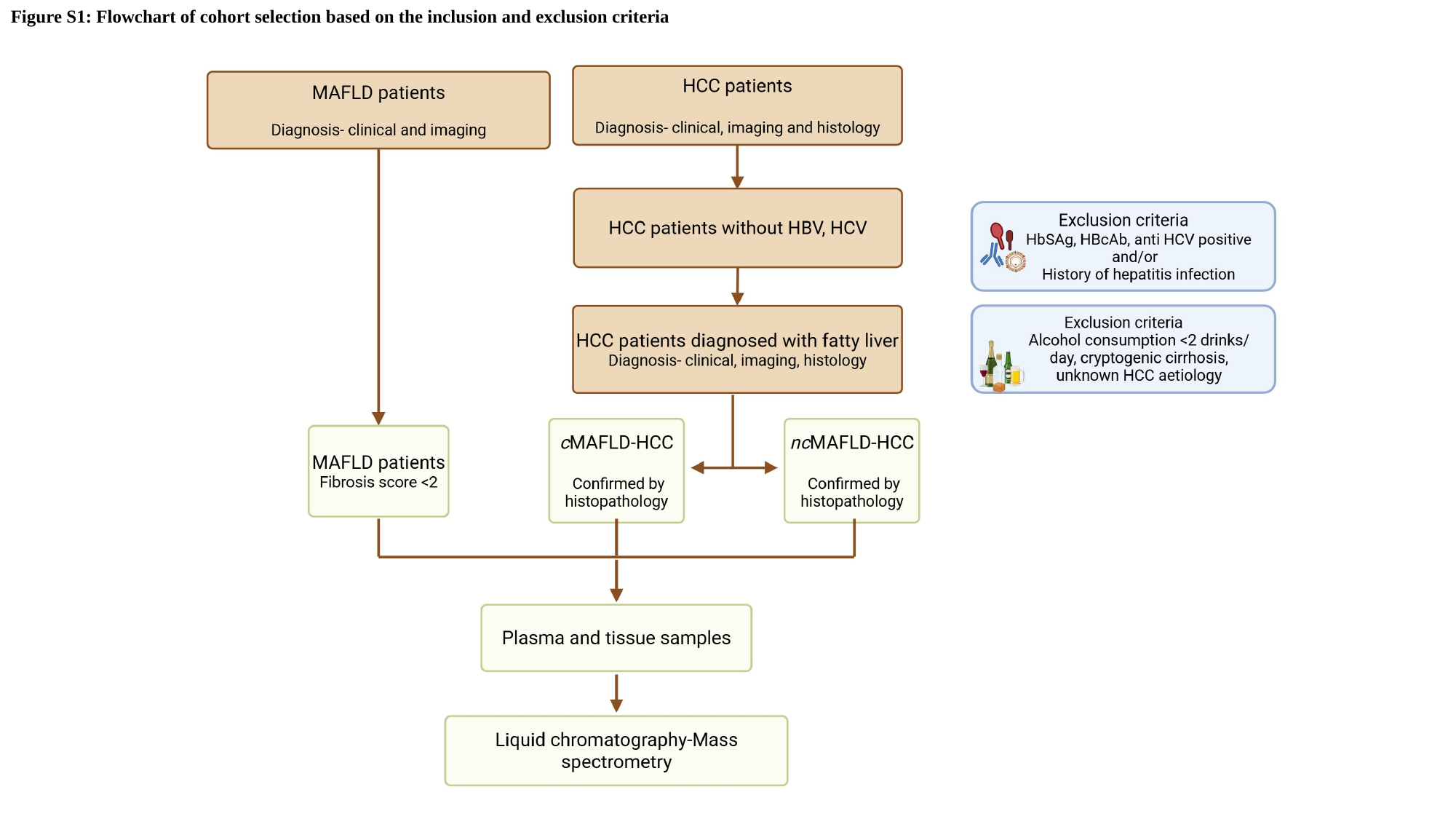

Figure S1: Flowchart of cohort selection based on the inclusion and exclusion criteria

## Slide 2
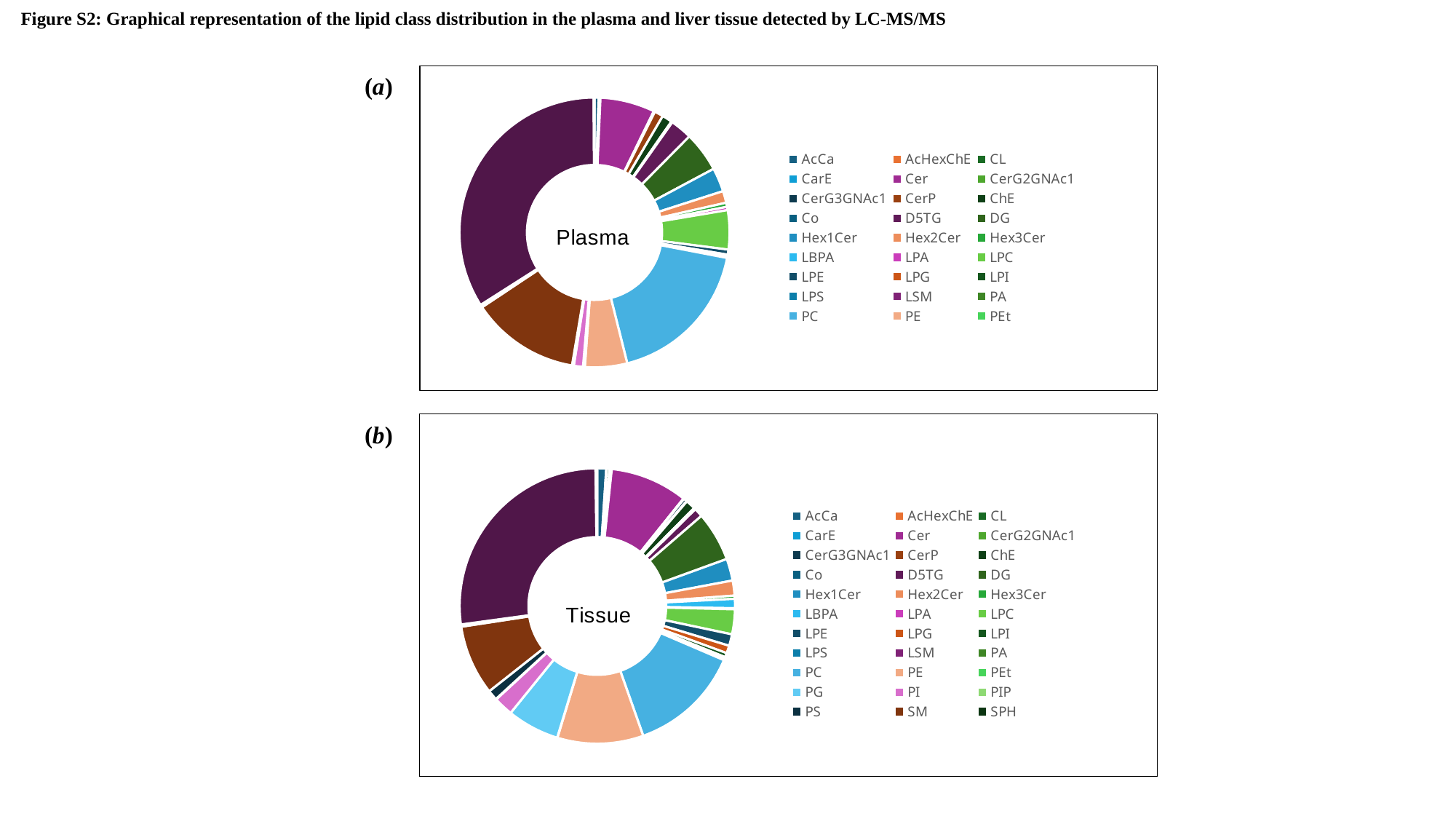

Figure S2: Graphical representation of the lipid class distribution in the plasma and liver tissue detected by LC-MS/MS
(a)
### Chart: Plasma
| Category | |
|---|---|
| AcCa | 0.4941757853865161 |
| AcHexChE | 0.03529827038475115 |
| CL | 0.0705965407695023 |
| CarE | 0.10589481115425343 |
| Cer | 6.530180021178962 |
| CerG2GNAc1 | 0.0 |
| CerG3GNAc1 | 0.1411930815390046 |
| CerP | 1.0589481115425343 |
| ChE | 1.200141193081539 |
| Co | 0.1411930815390046 |
| D5TG | 2.6120720084715847 |
| DG | 4.800564772326156 |
| Hex1Cer | 2.8591599011648428 |
| Hex2Cer | 1.4119308153900458 |
| Hex3Cer | 0.4941757853865161 |
| LBPA | 0.0 |
| LPA | 0.3882809742322626 |
| LPC | 4.694669961171902 |
| LPE | 0.6000705965407696 |
| LPG | 0.10589481115425343 |
| LPI | 0.1411930815390046 |
| LPS | 0.0 |
| LSM | 0.0 |
| PA | 0.1411930815390046 |
| PC | 18.072714436992587 |
| PE | 5.047652665019414 |
| PEt | 0.0 |
| PG | 0.21178962230850687 |
| PI | 1.0942463819272856 |
| PIP | 0.0 |
| PS | 0.21178962230850687 |
| SM | 12.989763501588422 |
| SPH | 0.10589481115425343 |
| ST | 0.21178962230850687 |
| TG | 33.921637839745856 |
| WE | 0.10589481115425343 |(b)
### Chart: Tissue
| Category | |
|---|---|
| AcCa | 1.0410641989589358 |
| AcHexChE | 0.0578368999421631 |
| CL | 0.31810294968189706 |
| CarE | 0.26026604973973394 |
| Cer | 9.080393290919607 |
| CerG2GNAc1 | 0.08675534991324466 |
| CerG3GNAc1 | 0.37593984962406013 |
| CerP | 0.0578368999421631 |
| ChE | 1.1278195488721805 |
| Co | 0.20242914979757085 |
| D5TG | 1.0410641989589358 |
| DG | 5.812608444187392 |
| Hex1Cer | 2.573742047426258 |
| Hex2Cer | 1.7640254482359745 |
| Hex3Cer | 0.37593984962406013 |
| LBPA | 1.098901098901099 |
| LPA | 0.1156737998843262 |
| LPC | 2.9207634470792367 |
| LPE | 1.3591671486408328 |
| LPG | 0.896471949103528 |
| LPI | 0.4916136495083864 |
| LPS | 0.0578368999421631 |
| LSM | 0.02891844997108155 |
| PA | 0.2891844997108155 |
| PC | 13.157894736842104 |
| PE | 10.09253903990746 |
| PEt | 0.02891844997108155 |
| PG | 6.130711393869289 |
| PI | 2.2845575477154423 |
| PIP | 0.0578368999421631 |
| PS | 1.1856564488143435 |
| SM | 8.212839791787161 |
| SPH | 0.26026604973973394 |
| ST | 0.0 |
| TG | 26.951995373048003 |
| WE | 0.20242914979757085 |

## Slide 3
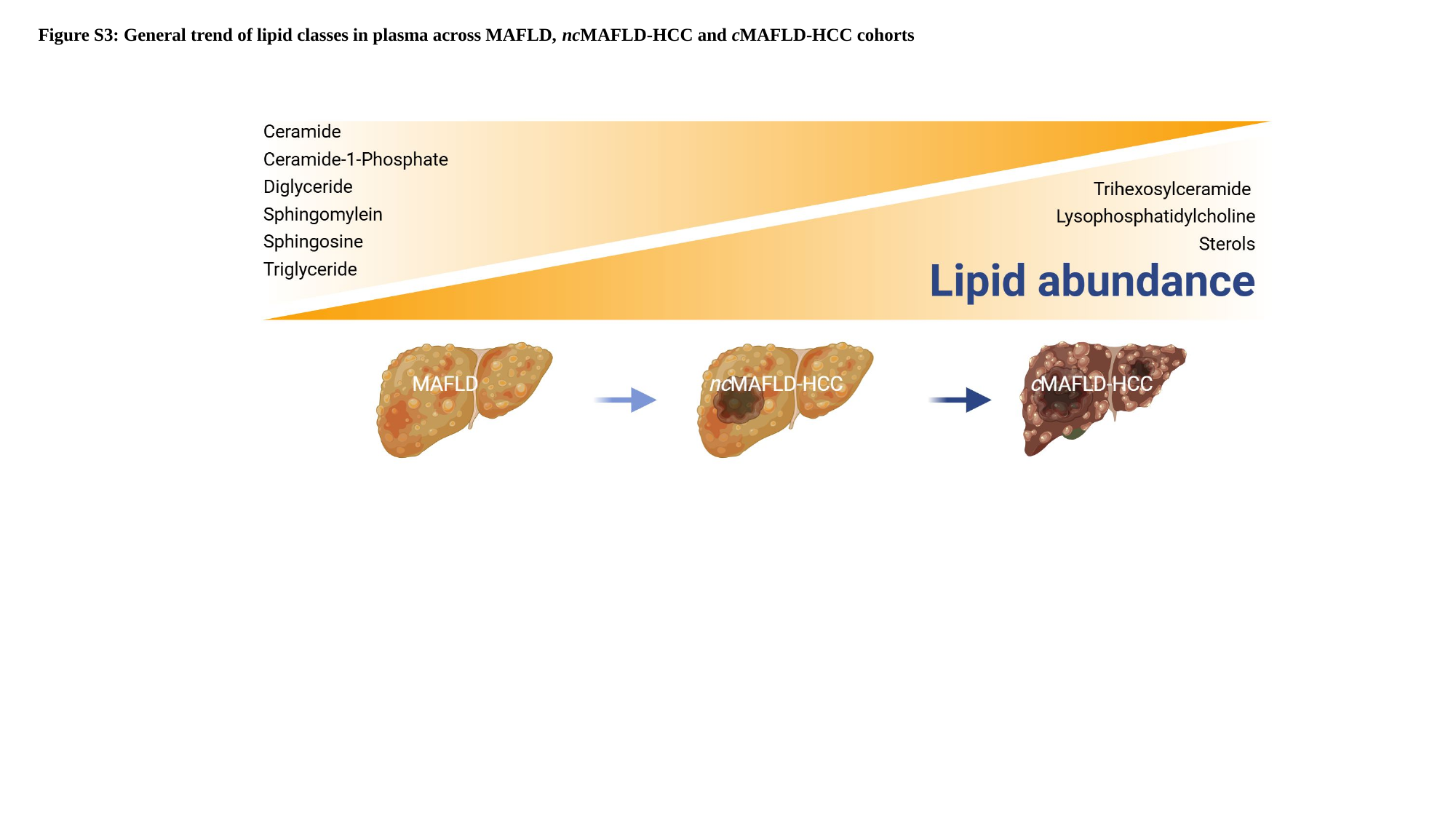

Figure S3: General trend of lipid classes in plasma across MAFLD, ncMAFLD-HCC and cMAFLD-HCC cohorts
